# Hypoxia- and MicroRNA-Induced Metabolic Reprogramming of Tumor-Initiating Cells

**DOI:** 10.3390/cells8060528

**Published:** 2019-06-01

**Authors:** Pit Ullmann, Martin Nurmik, Rubens Begaj, Serge Haan, Elisabeth Letellier

**Affiliations:** Molecular Disease Mechanisms Group, Life Sciences Research Unit, University of Luxembourg, L-4367 Belvaux, Luxembourg; ullmannpit@yahoo.com (P.U.); martin.nurmik@uni.lu (M.N.); rubens.begaj@uni.lu (R.B.); serge.haan@uni.lu (S.H.)

**Keywords:** colorectal cancer, cancer stem cell, tumor-initiating cell, stemness, hypoxia, microRNA, miR-210, lactate, metabolic reprogramming

## Abstract

Colorectal cancer (CRC), the second most common cause of cancer mortality in the Western world, is a highly heterogeneous disease that is driven by a rare subpopulation of tumorigenic cells, known as cancer stem cells (CSCs) or tumor-initiating cells (TICs). Over the past few years, a plethora of different approaches, aimed at identifying and eradicating these self-renewing TICs, have been described. A focus on the metabolic and bioenergetic differences between TICs and less aggressive differentiated cancer cells has thereby emerged as a promising strategy to specifically target the tumorigenic cell compartment. Extrinsic factors, such as nutrient availability or tumor hypoxia, are known to influence the metabolic state of TICs. In this review, we aim to summarize the current knowledge on environmental stress factors and how they affect the metabolism of TICs, with a special focus on microRNA (miRNA)- and hypoxia-induced effects on colon TICs.

## 1. Introduction

Tumor-initiating cells (TICs), also known as cancer stem cells (CSCs), have risen to great prominence over the past decade as potential key drivers of tumor development and progression. TICs were first described in hematological malignancies and were later found to be present in a number of different solid tumor types, including colorectal cancer (CRC) [1,2]. Despite extensive controversy with regards to their cellular origin and identification, TICs have been defined by their key functional features, such as their ability to self-renew and their capacity to replenish tumor heterogeneity via differentiation, both of which are features shared by tissue-specific stem cell populations. Due to these properties, TICs have the capacity to drive tumor initiation, maintenance, and progression. Most importantly, TICs are thought to evade conventional therapeutic options that are generally aimed at a population of differentiated cancer cells that are highly proliferative. Due to this, strategies aimed at specifically eradicating TICs might have a significant impact on the clinical outcome of CRC patients.

Cancer cells are known to adapt their metabolism in order to sustain high proliferation rates and survive in unfavorable environments with low oxygen and nutrient concentrations. In most cases, metabolic changes are driven by oncogenes or inactivated tumor suppressors, such as *MYC*, *TP53*, *AKT1*, or various Ras-related genes. Some metabolic changes, for example the shift towards glycolysis, seem to be universal characteristics of malignant tumor cells, while others, like changes in one-carbon or lipid metabolism, show tumor-specific patterns [1]. In this review, we aim to highlight the metabolic landscape of TICs and describe factors, such as hypoxia and microRNAs (miRNAs), that induce the metabolic reprogramming of TICs.

## 2. The Metabolic State of TICs

Recent evidence suggests that modifications of cellular metabolism have a tremendous impact on the regulation of stem cell and TIC properties [3]. The majority of differentiated cells oxidize glucose to carbon dioxide in the mitochondrial tricarboxylic acid (TCA) cycle, generating adenosine triphosphate (ATP), which helps to maintain cell homeostasis and cellular functions. In contrast, rapidly proliferating cancer cells undergo a metabolic shift, known as the Warburg effect, which is characterized by a switch towards active aerobic glycolysis [4]. In this process, glucose-derived carbons are diverted into anabolic pathways in order to build up biomass. Through this metabolic reprogramming, cancer cells accelerate ATP production and optimize the manufacture of building blocks for macromolecular synthesis [4].

While most studies agree that the majority of aggressive cancer cell populations predominantly rely on aerobic glycolysis, the metabolic state of colon TICs is still under debate. TICs seem to have different metabolic features according to the cancer type. In this regard, pancreatic TICs have been shown to rely on oxidative phosphorylation [5,6], highlighting the potential of metformin as a drug that may selectively target TICs in pancreatic tumors [6]. Cells resistant to different types of chemotherapy, in essence, TICs, are susceptible to the inhibition of mitochondrial metabolism [7]. In contrast, several other studies highlight that genetic, epigenetic, and environmental alterations of metabolic pathways promote the reprogramming of TICs, from normal mitochondrial oxidative phosphorylation (OXPHOS) towards increased glycolytic activity, which has been recognized as an important mechanism of cancer development [8]. In undifferentiated cells, including embryonic and pluripotent stem cells, the transcriptomic profile is more focused on glycolytic rather than oxidative metabolism, indicating that the pluripotent state of cells correlates with reduced mitochondrial respiration [9]. Similarly, *MYC*, the activation of which is one of the most common oncogenic events in all tumor types, plays a central role in metabolic reprogramming and is a particularly important target in TIC biology [10].

Importantly, distinct isolation approaches have led to the formation of opposing views with regards to the metabolic profile of colon TICs [11]. While CD133^+^CD44^+^Lgr5^+^ CRC cells have been shown to display a high activity of mitochondrial metabolism [12], other studies, in which the identification of TICs is based on an immature gene and protein expression profile rather than on specific surface markers, claim that stem cell-like colon TICs actively suppress oxidative phosphorylation by inhibiting the mitochondrial import of pyruvate [13]. In yet another approach, Vincent and colleagues have reported that CD133^+^ Colo205 cells express increased levels of glycolysis-related genes [14]. Along similar lines, CD133^+^ hepatocellular cells have been shown to display more active glycolysis over oxidative phosphorylation compared to CD133^-^ cells and their stemness characteristics can be reduced when glycolysis is inhibited [15]. Chen and colleagues reported that colon TICs actively downregulate several enzymes that are involved in the late steps of the TCA cycle, such as fumarate hydratase or malate dehydrogenase, which leads to the accumulation of early TCA cycle metabolites, such as citrate or α-ketoglutarate [16]. In our own studies, we have adopted a functional TIC isolation strategy by applying serum-free sphere culture conditions that specifically favor the growth of anchorage-independent TICs [17]. We could observe reduced TCA cycle activity and an increased production of lactate, which correlated with enhanced TIC self-renewal activity [18]. Our findings are in line with most studies, which seem to support the view that colon TICs display reduced OXPHOS and increased glycolytic activity.

In addition to active glycolysis, TICs also show other specific metabolic features. The mevalonate metabolic pathway produces cholesterol and coenzyme Q, as well as molecules involved in signal transduction, all of which are important in multiple cellular processes including cancer development and progression [19]. Evidence also exists to suggest that lipid metabolism may play a key role in TICs, as the inhibition of fatty acid synthesis via fatty acid synthase inhibitors, such as Cerulenin, has been shown to lead to a reduction in the expression of stemness markers in glioma TICs [20]. Other aspects such as increased glutamine metabolism have also been shown to significantly contribute to an aggressive TIC phenotype [11]. Many tumor cells are dependent on glutamine and thus display enhanced glutamine uptake rates and oxidative metabolism [21,22]. This process, commonly referred to as glutaminolysis, can be used to fuel the TCA cycle in case of glucose shortage [23]. As such, malignant CD44^+^ colon TICs, which have been recently shown to have higher glutamine levels compared to their non-tumorigenic CD44^-^ counterparts [24], may use glutamine as an additional energy source to sustain their self-renewal activity. Moreover, as glutamine dependency seems to negatively correlate with chemosensitivity [25,26], targeting the glutamine metabolism might help to overcome acquired drug resistance. Serine metabolism is another area which has recently risen in prominence with regards to cancer [27]. Future studies aimed at characterizing serine uptake in TICs and the role of serine metabolism during tumor initiation and progression may also be necessary in order to develop new strategies for targeting tumor metabolism.

## 3. Hypoxia Promotes Cancer Initiation and Progression

Another factor suggested to play a key role in the regulation and promotion of TICs and their metabolism is hypoxia. Due to excessive proliferation and abnormal blood vessel formation, most solid human tumors are irregularly vascularized and display local regions of hypoxia [28]. Accordingly, immunohistochemical analysis of 179 tumor specimens has revealed that hypoxia-inducible factor 1α (HIF1A) is frequently overexpressed in different cancer types, including breast, lung, and colon cancer [29]. Interestingly, different studies have shown that intratumoral hypoxia and the resulting upregulation of HIF1A and HIF2A signaling correlate with increased cancer patient mortality [30]. As such, primary tumors with high HIF1A protein expression have been linked to inferior disease-free and overall patient survival rates [31,32]. Along similar lines, Rasheed and colleagues have shown that elevated HIF1A levels are associated with vascular invasion and advanced TNM staging in rectal cancer patients [33], confirming that HIFs can be used as independent prognostic markers.

In addition to the well-established modulatory role that hypoxia plays in the immune response and invasive capacity of cancer cells, accumulating evidence suggests that hypoxia is also involved in the regulation of stem cell and TIC properties [34,35]. Interestingly, HIF1A signaling was shown to be essential for maintaining cell cycle quiescence of hematopoietic stem cells [36]. Furthermore, by using a murine knock-in model, Covello and colleagues were able to show that HIF2A directly regulates the pluripotency factor OCT4 [37], thereby unveiling the existence of a direct regulatory link between hypoxia, transcription factor signaling, and the expression of stem cell proteins. By using numerous cancer cell lines derived from different tumor types, Mathieu and colleagues have also shown that hypoxia is capable of driving the expression of various stemness factors, such as OCT4, NANOG, and SOX2 [38]. Accordingly, neuroblastoma [39], glioblastoma [40], breast cancer [41], prostate cancer [42], and CRC [43] were all shown to display an immature and stem cell-like phenotype under hypoxic culture conditions.

More specifically in the context of TICs, Soeda and colleagues have reported that a hypoxic microenvironment favors the expansion of aggressive CD133^+^ glioma stem cell-like cells [44]. Along similar lines, Wang and colleagues could observe that the pharmacological inhibition of HIF1A eliminates hematological TICs [45]. In addition, a study on breast cancer xenografts has shown that the generation of hypoxic tumor regions, via the administration of antiangiogenic agents, leads to a specific increase in TIC-like cell populations [46]. Li and colleagues were able to show that HIF2A expression correlates with both increased TIC properties and poor glioma patient survival [47], and a follow-up study further specified that HIF2A, in a feed-forward loop together with hypoxia-induced histone methyltransferase mixed-lineage leukemia 1 (MLL1), acts to drive TIC self-renewal and glioma initiation [48]. Interestingly, a recent study has also shown that the activity of demethylating ten-eleven translocation (TET) enzymes is O_2_-dependent and that a hypoxia-induced loss of TET activity triggers hypermethylation in several cancer-related gene promoters [49]. As the hypoxia-mediated deregulation of TET methylcytosine dioxygenase 1 (TET1) and TET3 has been shown to promote breast cancer TIC properties, such hypoxia-induced alterations of epigenetic controls can be considered as an important driving force of malignant tumor progression [50].

## 4. Metabolic Reprogramming of Cancer Cells Under Hypoxia

In addition to inducing molecular signaling events, hypoxia strongly affects the cellular metabolism: impaired oxidative phosphorylation, enhanced glycolytic activity, and increased production of mitochondrial reactive oxygen species (ROS) are only some of the known cellular characteristics induced by anaerobic conditions [51].

Indeed, reduced oxygen concentrations are known to potentiate the glycolytic phenotype of cancer cells in a HIF1A-dependent manner [52,53]. For instance, HIF1A-induced expression of pyruvate dehydrogenase kinase 1 (PDK1) has been found to phosphorylate pyruvate dehydrogenase (PDH) at a specific serine residue [54], resulting in decreased activity of the pyruvate dehydrogenase complex (PDC) [55,56]. As PDC is known to catalyze the conversion of pyruvate into acetyl-CoA, HIF1A/PDK1-mediated repression of its activity interferes with the TCA cycle and thus leads to reduced oxygen consumption and enhanced glycolysis [55,56]. Moreover, HIF1A induces the expression of glucose transporter (GLUT) 1 [57] and GLUT3 [58] as well as of many different glycolytic enzymes [59], resulting in both increased glucose uptake and more efficient glycolytic breakdown, respectively. Interestingly, in an O_2_-depleted environment, pyruvate is predominantly converted into lactate [22], thereby contributing to the shutdown of oxidative respiration under hypoxic conditions. In this context, lactate dehydrogenase A (*LDHA*) has been identified as a direct HIF1A target gene [60], illustrating again the importance of HIF1A in the mediation of a glycolytic phenotype.

## 5. MicroRNAs Regulate Metabolic Reprogramming

MicroRNAs are small non-coding RNAs composed of approximately 21–22 nucleotides, and were originally described in Caenorhabditis elegans [61]. They play an important role as vital posttranscriptional modulators of gene expression. Circulating miRNAs have been suggested to be potentially viable as a class of biomarkers [62], and a number of different miRNAs have been associated with the regulation of metabolism in cancer [63,64]. For instance, the loss of miR-143 in glioblastoma and CRC is thought to promote the expression of hexokinase 2, resulting in enhanced aerobic glycolysis [65,66]. Similarly, by counteracting the Warburg effect via the overexpression of miR124, miR-137, and miR-340, Wang and colleagues could demonstrate that the proliferation of CRC cells depends on their high glycolytic activity [67]. Another interesting miRNA with pleiotropic effects is miR-181a. In addition to sensitizing myeloid leukemia cells to chemotherapy- and natural killer (NK) cell-mediated killing [68], miR-181a was also shown to contribute to the Warburg effect by repressing phosphatase and tensin homolog (PTEN), thereby triggering a metabolic shift towards increased lactate production and CRC growth [69]. Interestingly, a large number of miRNAs also specifically control various tumorigenic processes in TICs by regulating their proliferation, aggressiveness, and metabolism (Table 1). As such, miR-122, a liver-specific miRNA, has been shown to inhibit TIC phenotypes by regulating glycolysis through PDK4 targeting [15]. Similarly, Wang and colleagues have recently demonstrated that MYC, via miR-33b induction, maintains glioblastoma TICs via the activation of mevalonate metabolism [70]. Targeting mevalonate metabolism, for example by means of statins, might therefore serve as a potential therapeutic strategy against TICs with limited toxicity. In a more general fashion, the miR-200c family and its roles in tumor progression have been widely described over the past few years [71,72]. Interestingly, miR-200c has been shown to enhance metabolic reprogramming via SIRT2 suppression, inducing pluripotency and stem cell functions in induced pluripotent stem cells (iPSCs) [73]. Whether this regulation of metabolism by the miR200c-SIRT2 axis is also operational in other types of stem-like cells, such as TICs, remains unknown. In addition to the miR200c family, the tumor suppressive role of let-7 miRNAs in a variety of cancer types has also been widely documented [74]. On top of that, let-7a has been shown to play an important role in reprogramming cancer metabolism by increasing both oxidative phosphorylation and glycolysis in triple-negative breast cancer and metastatic melanoma cell lines [75]. MicroRNAs also play a significant role in regulating other cell types in the tumor microenvironment. For example, miR-186a, a hypoxia-responsive microRNA that is an inhibitor of HIF1A-induced tumor proliferation in gastric cancer [76], has also been shown to be downregulated during fibroblast transformation, leading to an increased expression of GLUT1 and a glycolytic phenotype in cancer-associated fibroblasts (CAFs) [77]. All in all, miRNA-mediated reprogramming from oxidative phosphorylation towards aerobic glycolysis seems to affect both cancer cells and the tumor microenvironment, and can therefore be considered as an important driving force behind CRC progression.

**Figure 1:**
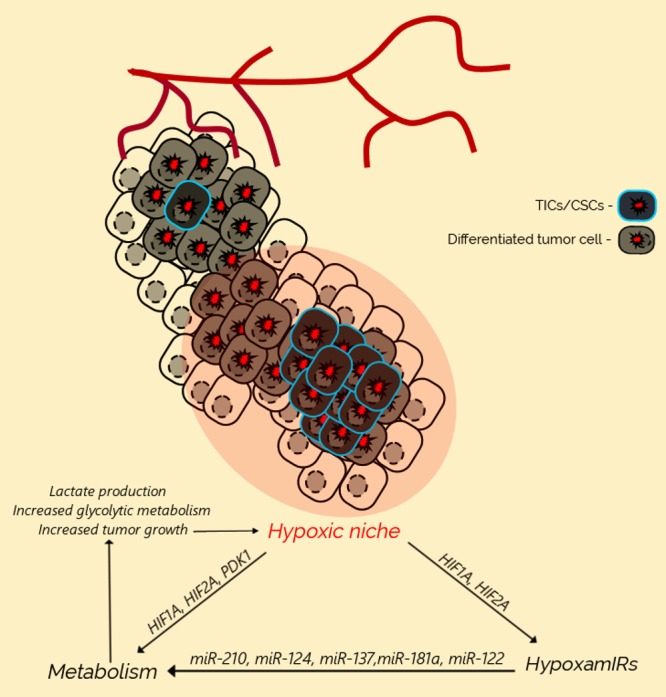
Hypoxia, miRNAs, and metabolism in the tumor niche. The local hypoxic niche in the tumor leads to both the activation of hypoxamiRs, such as miR-210, and extensive metabolic changes, via genes such as *HIF1A*, which in turn drive pro-tumorigenic characteristics such as lactate production and glycolytic metabolism, leading to tumor growth.

As hypoxia and metabolic changes are closely linked, it is important to also study the regulatory role of hypoxia-responsive miRNAs (HRMs) in the context of metabolic reprogramming (Figure 1). Specific miRNAs, such as miR-107 [95] and miR-22 [96], which affect the cellular response to hypoxia via *HIF1A* inhibition, can also be expected to have a large influence on the metabolism [63]. Likewise, *HIF2A* has also been shown to be regulated by miRNAs such as miR-30c-2-3p, miR-30a-3p, and miR-145 [97,98]. Keeping in mind that miRNAs, such as miR-145, have already been suggested to play a significant role in regulating tumor metabolism [99], it is likely that many miRNAs associated with the regulation of the *HIF* family play a significant role in regulating metabolism in tumor cells. In the same vein, the most prominent hypoxamiR, miR-210, is known to display multiple links to different metabolic processes, including autophagy and mitochondrial respiration [100]. For instance, miR-210 was shown to repress hypoxia-induced autophagy through the inhibition of *BNIP3*, thereby providing negative feedback to keep hypoxia-mediated effects in a physiological range [101]. On the other hand, it has been shown that miR-210 can also target *BCL2* [102], which could potentially lead to the induction of autophagy, via the disturbance of the BECN11/BCL2 complex.

Furthermore, besides affecting autophagy, miR-210 has been associated with the regulation of mitochondrial metabolism. By targeting two important respiratory chain components, namely iron-sulfur cluster assembly enzyme (ISCU) and heme A: farnesyltransferase cytochrome c oxidase assembly factor (COX10), miR-210 was shown to amplify the Warburg effect by repressing oxidative phosphorylation [103,104]. These findings were confirmed by Favaro and colleagues, who additionally reported that the miR-210-induced inhibition of ISCU leads to reduced aconitase and mitochondrial complex I activity, thereby triggering a metabolic shift towards enhanced glycolysis and increased cancer cell proliferation [105].

**Figure 2:**
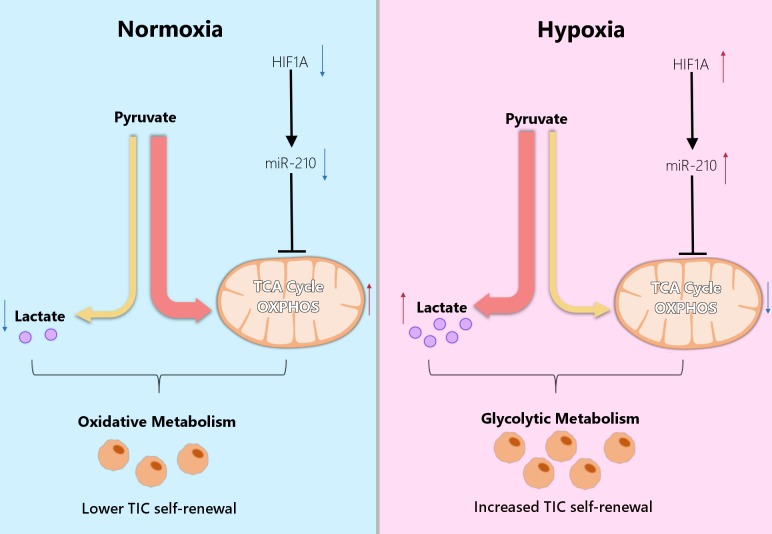
Hypoxia-responsive miR-210 drives the metabolic reprogramming and self-renewal activity of TICs. HIF1A-induced expression of miR-210-3p results in reduced TCA cycle activity and repressed oxidative phosphorylation under hypoxic conditions. The resulting metabolic shift leads to increased lactate production and drives cancer progression by promoting the self-renewal capacity of TICs.

HRMs, and in particular miR-210, are thus thought to further support the glycolytic nature of cancer cells [63]. In this context, the miR-210-induced inhibition of *ISCU* is known to repress both mitochondrial respiration and TCA cycle activity [103,105,106], and has been associated with breast cancer and head and neck squamous cell carcinoma progression [99]. Interestingly, our group has shown that a similar mechanism is involved in the metabolic reprogramming of colon TICs [18]. In this context, we were able to show that an increased expression of miR-210-3p and a reduced expression of ISCU correlate with CRC progression. Moreover, the stable overexpression of miR-210 in recently established CRC patient-derived spheroid cultures [17] resulted in significantly enhanced in vitro and in vivo TIC self-renewal activity [18]. By measuring the consumption/secretion rates of glucose and lactate, and by using a uniformly 13C-labeled glutamine tracer, we could show that miR-210 represses the TCA cycle activity of colon TICs by partially redirecting the intracellular flux of glycolytic pyruvate from oxidation in the TCA cycle to enhanced lactate production [18]. Importantly, we could demonstrate that miR-210-induced lactate secretion is largely responsible for the following observed effects. First, we were able to show that lactate stimulation leads to an increased self-renewal capacity of different colon TIC cultures. Secondly, a reduction in lactate production, via the pharmacological inhibition of LDHA, allowed us to block out the TIC-promoting effect of enhanced miR-210 and reduced ISCU expression [18]. Altogether, we could show that hypoxia-responsive miR-210, via the repression of ISCU, promotes the self-renewal capacity of colon TICs by triggering their metabolic reprogramming towards increased glycolysis and lactate production (Figure 2).

## 6. Lactate Acts as a TIC-Promoting Oncometabolite

Historically, lactate has long been considered as a mere waste product of aerobic glycolysis, however accumulating evidence now suggests that lactate can also be useful to cancer cells [22]. For instance, Wei and colleagues showed that the miR-181a-induced production of lactate results in enhanced cellular proliferation [69]. Similarly, high lactate levels were shown to promote an aggressive phenotype in breast cancer cells [107] and have been associated with a more stem cell-like gene expression profile in liver TICs [15,107]. By decreasing the extracellular pH, secreted lactate triggers metastasis via the degradation of the extracellular matrix (ECM) by pH-sensitive metalloproteinases [108,109]. It is important to note that intratumoral heterogeneity can also be observed on the metabolic level [23,110] and TIC populations of many different cancer types, including melanoma [111], osteosarcoma [112], liver [15], lung [113], and breast have been shown to display higher glycolytic activity than their non-TIC counterparts. The resulting increase in lactate further drives cancer progression by specifically promoting stem cell-like and tumorigenic properties [15,107]. Tumor hypoxia also further potentiates this glycolytic phenotype, thereby contributing to the overall metabolic reprogramming of TICs [11].

Our own experiments have shown that lactate stimulation promotes the self-renewal activity of colon TICs [18], further emphasizing the link between metabolic reprogramming and tumorigenic properties. Thus, targeting lactate metabolism might be an interesting approach for future anti-cancer therapies [114].

Accumulating evidence suggests that the high amounts of lactate that are produced during aerobic glycolysis can be beneficial for both the tumor cells and the tumor microenvironment (TME). While the precise definition and marker profile of CAFs still remains somewhat unclear [115], research indicates that lactate efflux from hypoxic CAFs may constitute an alternative energy source for adjacent cancer cells, thereby driving disease progression [116,117]. Experiments done using primary CAFs and tumor cell lines derived from ovarian cancer have shown that treating CAFs with tumor-conditioned media, or vice versa, can drastically increase lactate production in both cell types [118]. Recent studies indicate that exported lactate induces an inflammatory reaction, thereby attracting immune cells and leading to an increase in the production of growth factors and cytokines in the TME (30,31). Lactate is also known to contribute to the acidic pH of hypoxic TMEs [119], leading to the suppression of T-cells, which are known to be very pH-sensitive [120,121]. Thus, high lactic acid concentrations in the tumor microenvironment disable immune surveillance [119,122,123,124]. Taken together, lactate thus seems to exert several pro-tumorigenic functions by influencing both the TME and the tumor cells themselves, and can be considered as an important “oncometabolite” [8].

## 7. Clinical Targeting of Lactate Metabolism in TICs

Considering its role as a critical regulator of tumor development, targeting lactate metabolism might represent a promising approach for future anti-cancer therapies [114]. There are two ways to achieve a reduced secretion of lactate: either by targeting the monocarboxylate transporters (MCTs), which shuttle lactate out of the cell [125], or by inhibiting the lactate dehydrogenases, which convert pyruvate into lactate [126]. Both strategies will ultimately deprive developing tumors of a vital energy source. MCT1-4 transporters normally export the excessive levels of lactate produced [125], which is then used by the tumor cells as a source of energy to further promote cancer progression. Over the past few years, MCTs have been successfully targeted in pre-clinical studies of highly glycolytic malignant tumors [125]. In addition, LDHA inhibition, which is primarily expressed in cancer cells [127], has also emerged as an attractive potential method of clinical intervention. At the end of the glycolytic pathway, LDHA converts pyruvate to lactate, which is coupled with the oxidation of NADH to NAD^+^. The resulting elevated levels of LDHA, which are regulated by both HIF1A [60] and MYC [128], are known to be associated with an increased risk of invasion, metastasis, and patient death [126]. As a large majority of solid tumors are highly glycolytic, they display elevated levels of LDHA [129]. Recently, several studies have highlighted that high serum LDH levels can be associated with poor prognosis in different cancer types [130]. In addition to being a valuable predictive and/or prognostic marker, an increasing number of studies seem to indicate that LDHA may also be a viable therapeutic target. Selective knockdown studies, as well as pharmacological approaches using small-molecule inhibitors of LDHs, resulted in reduced in vitro and in vivo tumor growth in a variety of cancer types [113,127,131] due to the induction of apoptosis following increased ROS levels [132]. Interestingly, Xie and colleagues have also demonstrated that LDHA is vital for TIC survival and proliferation [113]. Moreover, our own research has also shown that the hypoxia-induced self-renewal of TICs can be reversed via LDHA inhibition [18]. As such, both lactate and LDHA can be considered as promising molecular targets for the development of glycolytic inhibitors for possible use in cancer therapy. LDHA inhibition, in particular, is currently being tested as a potential anti-cancer strategy in pre-clinical studies [114] and upcoming results could very well prove the clinical viability of LDHA inhibitors.

In conclusion, it has to be noted that hypoxia-induced metabolic reprogramming has a considerable impact on tumor initiation and progression. As intratumoral heterogeneity can be observed even at metabolic levels [133], and as miRNAs have been indicated to be capable of both inducing cell-to-cell heterogeneity alone [134] and via extracellular vesicle transfer to other cells [135], any current and future research initiatives in the field of anti-TIC drug development must take into account the differences in metabolism and miRNA expression between TICs and their non-tumorigenic counterparts. In this area, both oxidative stress-based therapies and the pharmacological inhibition of nitric oxide synthase are two promising avenues for TIC-specific therapies. Moreover, strategies that target the glucose metabolism of TICs have lately gained a lot of momentum [136] and, in this context, the anti-diabetic drug metformin has been suggested to specifically kill glycolytic CRC cells by modulating their glucose homeostasis [137]. As a number of papers have shown that the pharmacological inhibition of lactate production significantly represses TIC functionality, this also remains a viable potential avenue for tumor treatment to target this oncometabolite [18,113]. All in all, the clinical application of compounds that target key metabolic regulators, in both TICs and cancer in general, is a novel and promising field which will hopefully provide innovative treatment avenues for cancer patients in the future.

## Figures and Tables

**Table 1:** Tumor-initiating cells (TIC)-associated microRNAs (miRNAs) and their function.

miRNA	Target Gene	Role of miRNA	Tumor Type	Reference
miR-7	*KLF4, SETDB1*	Inhibits stemness and tumorigenesis by directly targeting KLF4, inhibits metastatic ability of breast TICs, reverses epithelial–mesenchymal transition (EMT) via SETDB1 targeting	Breast (Brain metastasis)	[78,79]
miR-21	*TGFBR2*	Induces stemness by activating the Wnt/β-catenin pathway through TGFBR2 downregulation	Colon	[80]
miR-33b	*MYC*	Regulates MYC via the RAS/ERK/miR33b pathway	Glioblastoma	[81]
miR-34a	*NOTCH1*	Controls symmetric/asymmetric cell division	Colon	[82]
miR-93	*HDAC8, TLE4*	Inhibits proliferation and colony formation	Colon	[83]
miR-125	*ALDH1A3, MCL1*	Regulates chemoresistance	Colon	[84]
miR-146a	*NUMB*	Controls symmetric/asymmetric cell division	Colon	[85]
miR-155	*TP53INP1*	Induces TIC-like phenotype by blocking the tumor suppressor gene TP53INP1	Liver	[86]
miR-193a	*PLAU, KRAS*	Inhibits tumorigenic potential	Breast, Colon, and Pancreas	[87]
miR-200c	*BMI1, SOX2*	Regulates chemoresistance and reduces tumorigenic capacity	Colon	[71,72]
miR-210	*ISCU, LDHA*	Promotes self-renewal of colorectal cancer (CRC) TICs by reducing tricarboxylic acid (TCA) cycle activity and enhancing lactate production.	Colon, Breast	[18,88]
miR-215	*BMI1, LGR5*	Promotes differentiation and inhibits stemness	Colon	[89,90]
miR-328	*ABCG2, MMP16*	Inhibits drug resistance and cell invasion	Colon	[91]
miR-451	*PTGS2, ABCB1*	Represses Wnt activation and chemoresistance	Colon	[92]
miR-520f	*SOX9*	Induces hypoxia-driven Sorafenib resistance by increasing the number of TIC-like cells	Liver	[93]
miR-1297	*SLC7A11*	Impairs cysteine uptake and glutathione production	Colon	[94]
